# Intermittent versus continuous enteral nutrition attenuates increases in insulin and leptin during short-term bed rest

**DOI:** 10.1007/s00421-020-04431-4

**Published:** 2020-07-10

**Authors:** Javier T. Gonzalez, Marlou L. Dirks, Andrew M. Holwerda, Imre W. K. Kouw, Luc J. C. van Loon

**Affiliations:** 1 Department for Health, University of Bath, Bath, UK; 2 Department of Human Biology, NUTRIM School of Nutrition and Translational Research in Metabolism, Maastricht University Medical Centre+ (MUMC+), Maastricht, The Netherlands

**Keywords:** Glucagon, Glucagon-like peptide-1, Glucose, Insulin, Insulin sensitivity, Metabolism

## Abstract

**Purpose:**

To compare endocrine responses to intermittent vs continuous enteral nutrition provision during short-term bed rest.

**Methods:**

Twenty healthy men underwent 7 days of bed rest, during which they were randomized to receive enteral nutrition (47%E as carbohydrate, 34%E as fat, 16%E as protein and 3%E as fibre) in a continuous (CONTINUOUS; *n* = 10; 24 h day^−1^ at a constant rate) or intermittent (INTERMITTENT; *n* = 10; as 4 meals per day separated by 5 h) pattern. Daily plasma samples were taken every morning to assess metabolite/hormone concentrations.

**Results:**

During bed rest, plasma leptin concentrations were elevated to a lesser extent with INTERMITTENT vs CONTINUOUS (iAUC: 0.42 ± 0.38 vs 0.95 ± 0.48 nmol L^−1^, respectively; *P* = 0.014) as were insulin concentrations (interaction effect, *P* < 0.001) which reached a peak of 369 ± 225 pmol L^−1^ in CONTINUOUS, compared to 94 ± 38 pmol L^−1^ in INTERMITTENT (*P* = 0.001). Changes in glucose infusion rate were positively correlated with changes in fasting plasma GLP-1 concentrations (*r* = 0.44, *P* = 0.049).

**Conclusion:**

Intermittent enteral nutrition attenuates the progressive rise in plasma leptin and insulinemia seen with continuous feeding during bed rest, suggesting that continuous feeding increases insulin requirements to maintain euglycemia. This raises the possibility that hepatic insulin sensitivity is impaired to a greater extent with continuous versus intermittent feeding during bed rest. To attenuate endocrine and metabolic changes with enteral feeding, an intermittent feeding strategy may, therefore, be preferable to continuous provision of nutrition.

This trial was registered on clinicaltrials.gov as NCT02521025.

## Introduction

Malnutrition during hospitalisation is a common occurrence (McWhirter and Pennington 1994) and is associated with poorer patient outcomes (Robinson et al. 1987) delayed discharge times, and an increase in associated costs (Reilly et al. 1988). When nutritional requirements cannot be met by normal eating due to either lack of consciousness, swallowing difficulties, or lack of appetite, then enteral nutritional support is advised, particularly when oral intake is likely to be insufficient (< 70% of requirements) for 3–7 days (Stroud et al. 2003; Singer et al. 2018). Enteral nutrition is preferred over parenteral nutrition due to lower infection risks (Braunschweig et al. 2001) and because it represents a more “physiological” mode of nutrition delivery (Stroud et al. 2003). For example, nutrients in the gastrointestinal tract are responsible for the incretin effect; a phenomenon whereby the peptides glucagon-like peptide-1 (GLP-1) and glucose-dependent insulinotropic polypeptide (GIP) stimulate insulin secretion. Accordingly, intravenous glucose does not raise GLP-1 concentrations above basal (Nielsen et al. 1985), whereas both oral ingestion (Nielsen et al. 1985; Chen et al. 2018) and enteral nutrition [either gastric or jejunal delivery (Luttikhold et al. 2016)] potently raise plasma postprandial GLP-1 concentrations above fasting values. Importantly, the incretin effect has a central role in metabolic control; responsible for the majority (up to 70%) of postprandial insulin secretion in healthy humans (Nauck et al. 2004; Nauck and Meier 2016).

Metabolic complications are common with nutritional therapy and the prevalence of insulin resistance in patients with enteral tube feeding is ~ 33% (Woolfson et al. 1976), which could be due to a number of factors including muscle disuse (Dirks et al. 2016), injury/illness-associated stress (Woolfson et al. 1976) and/or the endocrine disruption due to enteral nutrition delivery pattern and mode (Luttikhold et al. 2016; Stoll et al. 2012). In intensive care, it is most commonly recommended to provide enteral nutrition in a continuous pattern, rather than an intermittent pattern that better mimics feeding patterns in normal daily life (Singer et al. 2018; NICE 2006). There is little information on the role of enteral feeding patterns on endocrine and metabolic responses in vivo in humans. The decline in peripheral insulin sensitivity during bed rest with enteral nutrition appears to be independent of the pattern (intermittent vs continuous) of enteral nutrition delivery in humans (Dirks et al. 2019a). However, it is unknown whether feeding pattern alters other aspects of metabolism or endocrine function.

Alongside evidence that exogenous GLP-1 improves glycaemic control in healthy and critically ill humans (Deane et al. 2009; Gutniak et al. 1992; D'Alessio et al. 1994), it has been suggested that endocrine alterations (including GLP-1) may be a key mechanism underlying the metabolic responses to differing delivery modes and patterns of enteral nutrition (Luttikhold et al. 2016; Stoll et al. 2012). Alongside GLP-1, other peptide hormones, such as ghrelin, glucagon and leptin are also responsive to feeding, and all play important roles in glucose metabolism, insulin sensitivity and appetite regulation (Kojima and Kangawa 2005; Morton and Schwartz 2011; Friedman 2014; Troke et al. 2014; Sandoval and D'Alessio 2015). In the early phase of enteral feeding (first 4 h), an intermittent pattern produces a divergent insulin and gut hormone response compared to a continuous pattern (Chowdhury et al. 2016). Non-randomized trials indicate that intermittent feeding over 3 days suppresses appetite ratings and plasma ghrelin concentrations and raises plasma leptin and glucagon concentrations compared to placebo (Stratton et al. 2008), whereas continuous feeding does not suppress appetite or alter plasma glucagon concentrations compared to placebo (Stratton et al. 2003). To date, however, there are no direct comparisons of intermittent vs continuous enteral tube feeding on peptide responses over a time-period when enteral nutrition is typically advised (i.e. ~ 5–7 days).

An opportunity arose to address whether enteral feeding pattern alters endocrine responses during 7 days of bed rest by making use of plasma samples collected as part of a wider project examining muscle metabolism and mitochondrial capacity (Dirks et al. 2019a, b). Blood samples and appetite ratings were taken each morning throughout 7 days of bed rest with enteral nutrition delivered in an intermittent vs continuous pattern. Accordingly, the aim of this study was to assess the effects of an intermittent pattern of enteral nutrition during 7 days of bed rest on endocrine (insulin, glucagon, GLP-1, ghrelin, and leptin) responses, compared to current standard practice of continuous enteral nutrition. We also aimed to characterise the relationship between endocrine responses, insulin sensitivity, and appetite. We hypothesised that intermittent feeding may increase plasma glucagon and ghrelin concentrations alongside increases in appetite ratings, whilst decreasing insulin concentrations, when compared to continuous feeding.

## Materials and methods

### Study design

The data presented in the current manuscript are part of a larger study on muscle metabolic responses to enteral feeding patterns during bedrest (Dirks et al. 2019a). Where data have previously been reported, this is clearly stated and cited. All endocrine data presented in this manuscript have not been reported previously. After screening, 20 participants completed baseline testing and began a control period comprising 7 days of standardised nutrition. On day 7 of the control period, participants underwent body composition testing and a hyperinsulinemic–euglycemic clamp to determine whole-body insulin sensitivity. On the same evening, participants arrived at the University for insertion of a nasogastric tube and remained at the University overnight. The following morning at 08:00, a 7-day period of bed rest began. During this period, participants were randomized to receive enteral nutrition in either an intermittent (INTERMITTENT; *n* = 10; 4 meals per day) or continuous (CONTINUOUS; *n* = 10; 24 h day^−1^ at a constant rate) pattern. After 7 days of bed rest, the hyperinsulinemic–euglycemic clamp was repeated to determine changes in whole-body insulin sensitivity.

In order to characterise the acute responses with temporal resolution, one additional participant with characteristics representative of study population (age: 28 years; stature: 1.86 m; body mass: 93 kg) completed two, 24-h periods of bed rest with nasogastric feeding in either an intermittent (4 meals per day) or continuous pattern, with a 7-day washout period. Blood was sampled hourly to characterise the initial diurnal responses to the protocol.

### Participants

The present study recruited young healthy men (age range for recruitment: 18–35 years), on the basis that this would allow for an understanding of the effects of feeding pattern per se, without the potential confounding of interactions with injuries/illnesses. Participant’s characteristics are presented in Table 1 [as previously reported (Dirks et al. 2019a)]. All participants were informed of the nature and risks of the experiment prior to taking part before being informed, written consent was obtained. Prior to participation, participants also completed a general health questionnaire and medical screening to determine their eligibility to participate. Exclusion criteria included the following: a body mass index below 18.5 or above 30 kg m^−2^; a family history of deep vein thrombosis, type 2 diabetes (defined by HbA1c > 7.0%); any back, knee or shoulder complaints that could be contraindicative of bed rest; and participation (in the 6 months prior to the study) in resistance-type exercise training. During screening, a blood sample was obtained in the fasted state and resting metabolic rate was determined using a ventilated hood. The current study was approved by the Medical Ethical Committee of Maastricht University Medical Centre^+^ (registration number MEC 15-3-035) in accordance with the Declaration of Helsinki.

**Table 1:** Participant characteristics

	INTERMITTENT (*n* = 10)	CONTINUOUS (*n* = 10)
Age (year)	27 ± 4	24 ± 4
Stature (m)	1.81 ± 0.09	1.79 ± 0.08
Body mass (kg)	77.5 ± 16.2	77.3 ± 16.1
Body mass index (kg m^−2^)	23.5 ± 4.0	24.0 ± 3.2
HbA_1c_ (%)	5.2 ± 0.3	5.2 ± 0.5
Body fat (%)	22.9 ± 5.9	22.3 ± 3.8

Data previously reported (Dirks et al. 2019a). HbA_1c_, glycated haemoglobin

*INTERMITTENT* intermittent enteral nutrition pattern, *CONTINUOUS* continuous enteral nutrition pattern

### Bed rest

Participants underwent a 7-day period of strict bed rest to mimic the effects of hospitalization. On day 1 of bed rest, at 08:00, participants began the bed rest procedure during which they were not permitted to leave the bed for 7 days. Participants were woken at 07:30 and lights were switched off at 23:30 daily. During daytime, participants were permitted to use a pillow and slight elevation of the bed in order to perform daily activities. Washing and all sanitary activities were performed in bed. Participants were constantly monitored by members of the research team.

### Nutritional intake

During screening, resting metabolic rate (RMR) was determined via indirect calorimetry using an open-circuit ventilated hood system [Omnical, Maastricht University, Maastricht, the Netherlands; (Schoffelen et al. 1985)]. During the 7-day control period prior to bed rest, and during the bed rest period, all nutrition was provided by the research team and dietary intake was standardised to achieve energy balance based on RMR multiplied by either 1.60 (control period prior to bed res) or 1.35 (for bed rest). The macronutrient composition of the diet was identical during both the control period prior to bed rest and the bed rest period.

During bed rest, enteral nutrition was provided via a nasogastric tube (Flocare© PUR tube Enlock, Ch8, 110 cm, Nutricia Advanced Medical Nutrition, the Netherlands). Correct placement of the tube in the stomach was confirmed by pH assessment immediately following insertion and on every morning during bed rest. A standard enteral nutrition product (Nutrison Multi Fibre, Nutricia Advanced Medical Nutrition) was employed, providing carbohydrates (47% energy) fat (34% energy), protein (16% energy) and fibre (3% energy). During INTERMITTENT, participants received nutrition in four daily boluses, administered at 08:00 (30% of daily energy intake; ~ 720 kcal over 30 min), 13:00 (30% of daily energy intake; ~ 720 kcal over 30 min), 18:00 (30% of daily energy intake; ~ 720 kcal over 30 min) and 23:00 (10% of daily energy intake; ~ 240 kcal over 10 min) at a rate of 25 mL min^−1^, with the first meal administered on the morning of the first day of bed rest. During CONTINUOUS, participants received nutrition in a continuous pattern via a Flocare© Infinity enteral feeding pump (Nutricia Advanced Medical Nutrition) at a constant rate (~ 100 mL h^−1^, depending on energy requirements, equating to a mean intake of ~ 100 kcal h^−1^). Continuous feeding began at 00:00 on the evening before bed rest and ended at 00:00 on the evening of day 7 to allow for the final measures to be determined in a fasted state. Nasogastric tubes were removed at 00:00 on the evening of day 7 in both groups.

### Body composition

Height was determined using a stadiometer, and body mass was determined with participants wearing minimal clothing. Body composition was determined by dual-energy X-ray absorptiometry (DXA; Hologic, Discovery A, QDR Series, Bradford, MA, USA) The software package Apex v 4.0.2 was used to determine whole-body fat mass.

### Hyperinsulinemic–euglycemic clamp

Whole-body insulin sensitivity was assessed by a hyperinsulinemic–euglycemic clamp on the days immediately before and after bed rest as previously described (Dirks et al. 2016). Briefly, an intravenous cannula was inserted retrogradely into a dorsal hand vein, with the hand kept in a heated box (60 °C) for sampling of arterialised blood. In the contralateral arm, an intravenous cannula was inserted into an antecubital vein for infusion of 20% glucose (Baxter B.V., Utrecht, Netherlands) and insulin (40 mU m^−2^ min^−1^; Novorapid, Novo Nordisk Farma, Alphen aan den Rijn, the Netherlands). Blood was sampled every 5 min throughout the 2.5-h clamp to determine glucose concentration (ABL800 FLEX; Radiometer Medical, Brønshøj, Denmark). Glucose infusion rate (GIR) was altered in order to maintain euglycemia at 5.0 mmol L^−1^, and average GIR of the last 30 min was used as a measure of peripheral insulin sensitivity.

### Blood sampling and analysis

Blood was sampled via venepuncture prior to the control period, and then at 08:00 on each morning of bed rest. Since the CONTINUOUS group were constantly in a fed state, all blood samples during bed rest were taken in a “postprandial” state in this group, vs a fasted state in the INTERMITTENT group. In order to ascertain an effect of feeding pattern independent from feeding status, a final blood sample was collected at 08:00 (in the fasted state for both groups) on the day after bed rest (day 8) prior to the hyperinsulinemic-euglycemic clamp. Samples were collected in EDTA-coated tubes and immediately centrifuged at 1000*g* for 10 min at 4 °C. Plasma was divided into aliquots, snap frozen in liquid nitrogen and stored at − 80 °C until subsequent determination of glucose (GLUC3, reference 05168791 190, Roche; detection limits; 0.11–41.6 mmol L^−1^), glycated haemoglobin (determined in 4 mL venous blood by high-performance liquid chromatography; Bio-Rad Diamat, Munich, Germany) insulin (Immunologic, reference 12017547 122, Roche; detection limits; 1.39–6945 pmol L^−1^), GLP-1_Total_ (Epitope Diagnostics Inc. CA, USA; detection limits; 0.6–54 pmol L^−1^) ghrelin (EMD Millipore, Germany; detection limits; 50–5000 pg mL^−1^) glucagon (Mercodia AB, Sweden; detection limits; 0.024–100,000 pmol L^−1^) leptin (Mercodia AB, Sweden). Intra- and inter-assay co-efficients of variation were all < 8%.

### Appetite ratings

Appetite ratings were determined using 100 mm visual analogue scales (Flint et al. 2000) with descriptors anchored at each end describing the extremes (e.g. “I am not hungry at all” versus “I have never been more hungry”). Participants rated their appetite by placing a vertical mark across each line on paper, and participants were not permitted to refer to their previous ratings when completing the scales. Scales were analysed by measuring the horizontal distance from the left-had anchor to the point marked by the participant.

### Statistics

The sample size for the study was based on the primary outcome of glucose infusion rate as reported in the parent manuscript (Dirks et al. 2019a). However, this sample size should also be sufficient to detect meaningful differences in endocrine responses based on data reported by Stoll et al. (Stoll et al. 2012), whereby INTERMITTENT resulted in a plasma GLP-1 concentrations of 43 ± 11 pmol L^−1^, compared to 32 ± 3 pmol L^−1^ with CONTINUOUS. Using this effect size (*d* = 1.36), ten participants in each group should provide a power of 0.82 with a two-tailed *α*-level of 0.05 in a between-group design (Faul et al. 2007).

Data are expressed as mean ± SD in text and tables and mean ± 95% CI in figures. Data were analysed using Prism v7 (GraphPad Software, San Diego, CA, USA) and log-transformed if appropriate, prior to analysis (determined by Shapiro–Wilk normality test). Baseline differences between groups were assessed using independent *t* test. Changes over time were assessed by repeated measures ANOVA, with time (days 0–7 or pre- vs post-bed rest) as the within-subjects factor and group (INTERMITTENT vs CONTINUOUS) as the between-subjects factor. The incremental area under the curve (iAUC) for plasma peptide concentrations over time (above baseline for GLP-1, glucagon and leptin; below baseline for ghrelin), was calculated using the trapezoidal method to use as a summary statistic. Between group differences (INTERMITTENT vs CONTINUOUS) in iAUC, and pre- to post-bed rest change in fasting variables (glucose infusion rate, plasma glucose and hormone concentrations) were analysed by independent *t* tests. Relationships between variables were analysed by Pearson correlation coefficients when normally distributed, or Spearman correlation coefficients when non-normally distributed (determined by Shapiro–Wilk normality test). All *P* values were corrected for multiple comparisons using the Holm–Sidak adjustment. A *P* value of ≤ 0.05 was used to determine statistical significance.

## Results

### Acute endocrine responses to modulation of 24-h enteral nutrition pattern

**Fig. 1:**
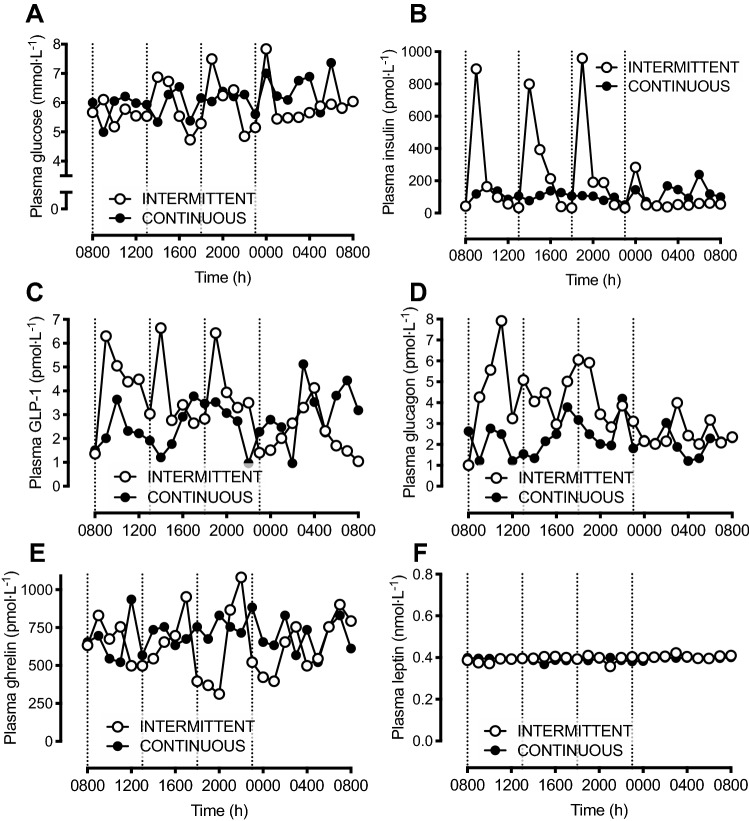
Plasma glucose (**a**), insulin (**b**), GLP-1 (**c**), glucagon (**d**), ghrelin (**e**) and leptin (**f**) concentrations during 24 h of bed rest in a representative participant (*n* = 1), with enteral nutrition provided in either an intermittent (INTERMITTENT) or continuous (CONTINUOUS) pattern. Dashed vertical lines indicate when nutrition was provided in the intermittent condition. *GLP-1* glucagon-like peptide-1

The acute (24-h) endocrine responses to intermittent vs continuous enteral feeding are displayed in Fig. 1, using data from an additional participant (*n* = 1). Plasma glucose concentrations followed a relatively similar pattern with intermittent vs continuous feeding (Fig. 1a), whereas plasma insulin concentrations varied markedly more with intermittent, compared to continuous feeding (Fig. 1b), increasing ~ ninefold following each main bolus feed. At the 24-h timepoint, plasma insulin concentrations were comparable between intermittent and continuous feeding patterns. Plasma GLP-1 concentrations increased > fourfold within 1 h after each main meal during INTERMITTENT, returning to basal concentrations following the overnight period (Fig. 1c). During CONTINUOUS, plasma GLP-1 concentrations displayed less variability (Fig. 1c). Similarly, plasma glucagon concentrations (Fig. 1d) fluctuated more widely with INTERMITTENT vs CONTINUOUS, increasing > sevenfold from basal with the first meal during INTERMITTENT, whereas during CONTINUOUS plasma glucagon concentrations did not rise more than twofold above basal values at any time point. By 24 h, plasma glucagon concentrations were comparable to baseline values during both INTERMITTENT and CONTINUOUS. Plasma ghrelin concentrations (Fig. 1e) decreased with feeding during INTERMITTENT, from ~ 640 to 312 pmol L^−1^, whereas plasma ghrelin concentrations did not decrease below 522 pmol L^−1^ at any time point during CONTINUOUS (Fig. 1e). Plasma leptin concentrations, however, remained stable over 24 h and did not differ between INTERMITTENT vs CONTINUOUS feeding (Fig. 1f).

### Plasma glucose, insulin and glucagon responses during 7 days of bed rest

**Fig. 2:**
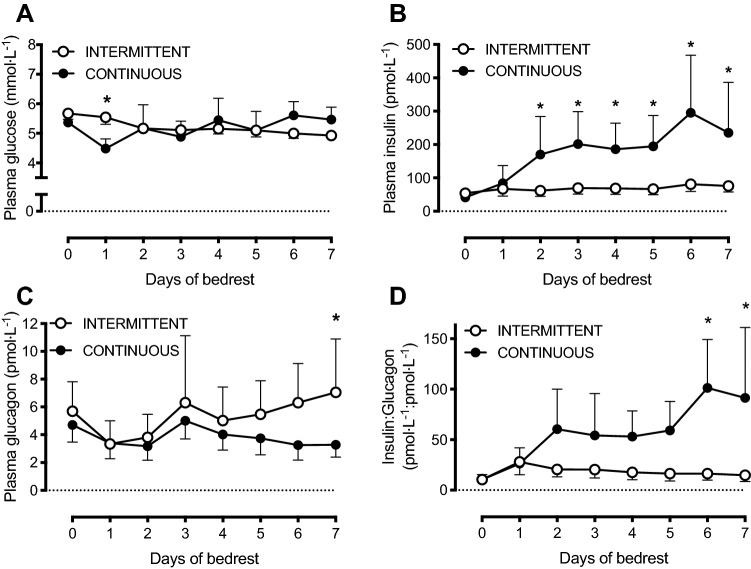
Plasma concentrations of glucose (**a**), insulin (**b**), glucagon (**c**) and the insulin-to-glucagon ratio (**d**) before (day 0) and during 7 days of bed rest, and with enteral nutrition provided in either an intermittent (INTERMITTENT; *n* = 10) or continuous (CONTINUOUS; *n* = 10) pattern. Data are means ± 95% CI. *difference between INTERMITTENT and CONTINUOUS, *P* < 0.05

**Fig. 3:**
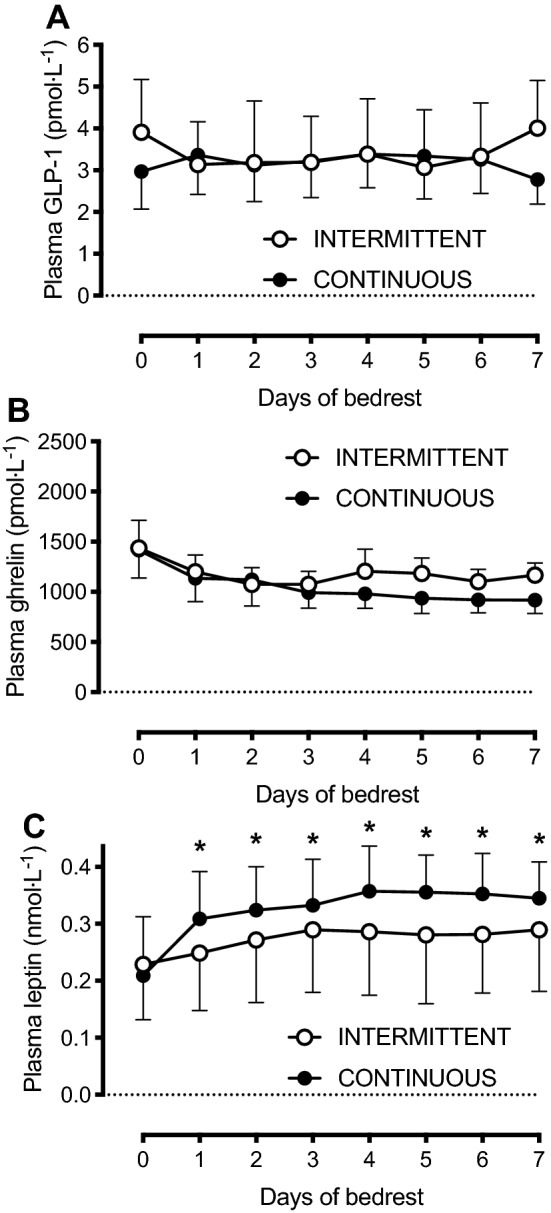
Plasma concentrations of GLP-1 (**a** glucagon-like peptide-1), ghrelin (**b**), and leptin (**c**) concentrations before (day 0) and during 7 days of bed rest, and with enteral nutrition provided in either an intermittent (INTERMITTENT; *n* = 10) or continuous (CONTINUOUS; *n* = 10) pattern. Data are presented as group means ± 95% CI. *P < 0.05 for INTERMITTENT vs CONTINUOUS

At baseline, no differences were detected between INTERMITTENT vs CONTINUOUS in plasma glucose, insulin, GLP-1, glucagon, ghrelin, or leptin concentrations, nor insulin-to-glucagon ratio (all *P* > 0.05; Figs. 2, 3). Whilst a modest difference in plasma glucose concentrations was observed after day 1 of bed rest with NTERMITTENT vs CONTINUOUS, (interaction effect, *P* < 0.001); thereafter, plasma glucose concentrations did not differ between conditions for the remainder of the 7 days of bed rest (Fig. 2a).

Plasma insulin concentrations did not differ between INTERMITTENT vs CONTINUOUS for the first day of bed rest. Thereafter, plasma insulin concentrations remained stable in INTERMITTENT, but began to rise in CONTINUOUS (interaction effect, *P* < 0.001; Fig. 2b), reaching peak concentrations of 94 ± 38 pmol L^−1^ in INTERMITTENT, compared to 369 ± 225 pmol L^−1^ in CONTINUOUS (*P* = 0.001).

Plasma glucagon concentrations began to decrease during the first 2 days of bed rest in both groups (time effect, *P* = 0.002), but towards day 7 of bedrest, plasma glucagon concentrations began to rise in the INTERMITTENT group only (interaction effect, *P* = 0.042; Fig. 2c).

The plasma insulin-to-glucagon ratio remained stable during bed rest with INTERMITTENT, but increased with CONTINUOUS (interaction effect, *P* < 0.001) such that by days 6 and 7, the plasma insulin-to-glucagon ratio was lower with INTERMITTENT vs CONTINUOUS (Fig. 2d; *P* < 0.05).

### Plasma GLP-1, ghrelin and leptin responses during 7 days of bed rest

Plasma GLP-1 concentrations (Fig. 3a) were not altered during bed rest compared to baseline values (time effect, *P* = 0.93). The delivery pattern of enteral nutrition, however, did alter the plasma GLP-1 response over time (interaction effect, *P* = 0.024), although following adjustment for multiple comparisons, none of the differences between feeding patterns at any time point remained significant (Fig. 3a).

Compared to baseline, plasma ghrelin concentrations decreased during bed rest (time effect, *P* < 0.001), to a similar extent in INTERMITTENT vs CONTINUOUS groups (interaction effect, *P* = 0.79; Fig. 3b).

Plasma leptin concentrations increased during bed rest, compared to baseline (time effect, *P* < 0.001) but to a lesser extent in the INTERMITTENT vs CONTINUOUS group (interaction effect, *P* = 0.001; Fig. 3c). Moreover, the leptin iAUC was lower with INTERMITTENT vs CONTINUOUS (0.95 ± 0.48 vs 0.42 ± 0.38 nmol L^−1^, respectively; *P* = 0.014).

### Appetite ratings during bed rest

**Fig. 4:**
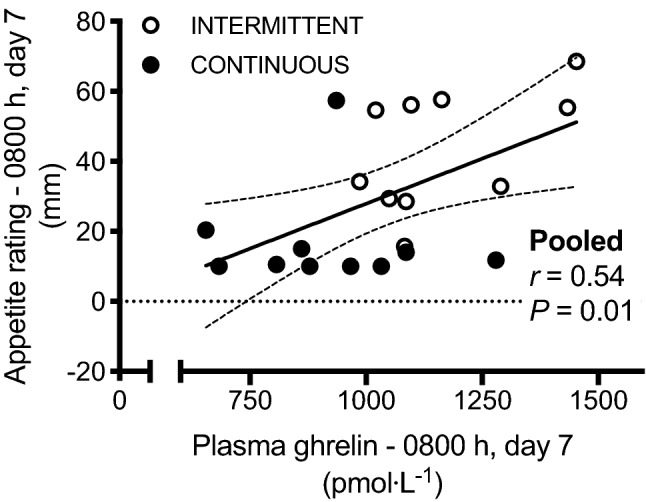
Relationship between plasma ghrelin concentration and appetite ratings during day 7 of bed rest conducted with enteral nutrition provided in either an intermittent (INTERMITTENT) or continuous (CONTINUOUS) pattern

During bed rest, appetite ratings were higher in the INTERMITTENT vs CONTINUOUS group (group effect, *P* = 0.01). Following adjustment for multiple comparisons, the INTERMITTENT group reported higher appetite ratings at both 0800 and 1800 h on day 7 only (Table 2). Moreover, appetite ratings on day 7 positively correlated with plasma ghrelin concentrations sampled at the same time point (Fig. 4). Appetite ratings did not correlate with either plasma glucagon or leptin concentrations (both *P* > 0.05).

**Table 2:** Appetite ratings during 7 days of bedrest with enteral nutrition provided in either an intermittent or continuous pattern

Day	Time	Appetite rating (mm)		INTERMITTENT vs CONTINUOUS Adjusted *P* value
		INTERMITTENT (*n* = 10)	CONTINUOUS (*n* = 10)	
Day 1	0800 h	39 ± 24	18 ± 14	0.162
	1800 h	38 ± 24	23 ± 18	0.783
Day 4	0800 h	39 ± 22	21 ± 18	0.376
	1800 h	40 ± 22	19 ± 14	0.115
Day 7	0800 h	41 ± 16	18 ± 15	0.029
	1800 h	46 ± 21	16 ± 14	0.009

*INTERMITTENT* intermittent enteral nutrition pattern, *CONTINUOUS* continuous enteral nutrition pattern

### Changes in fasting plasma glucose and regulatory hormones following bedrest

**Fig. 5:**
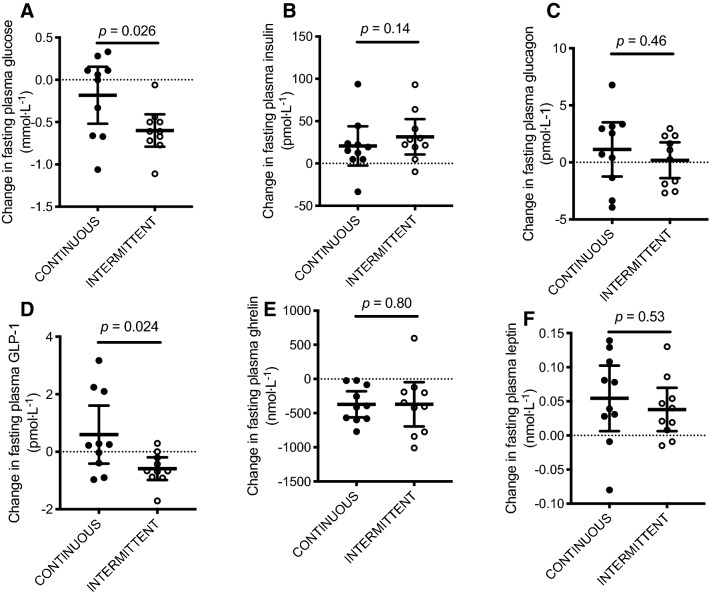
Change in fasting plasma glucose (**a**) insulin (**b**), glucagon (**c**), GLP-1 (**d**), ghrelin (**e**) and leptin (**f**) concentrations after 7 days of bed rest with enteral nutrition provided in either an intermittent (INTERMITTENT) or continuous (CONTINUOUS) pattern. GLP-1, glucagon-like peptide-1. Individual data are presented (*n* = 10 per group) alongside group means ± 95% CI

Fasting plasma GLP-1 concentrations decreased from pre-to-post 7 days of bed rest with INTERMITTENT versus CONTINUOUS (*P* = 0.024; Fig. 5d). In contrast, fasting glucagon, ghrelin and leptin concentrations were unaltered by enteral feeding pattern (all *P* > 0.05 for; Fig. 5a–c, e, f).

### Insulin sensitivity

**Fig. 6:**
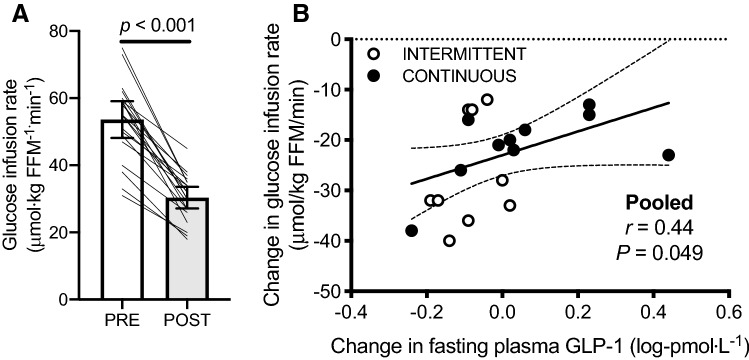
Glucose infusion rate during a hyper-insulinemic, euglycemic clamp before (PRE) and after (POST) 7 days of bed rest with enteral nutrition (**a**), as previously reported (Dirks et al. 2019a). Relationship between the change in fasting plasma GLP-1 concentration and glucose infusion rate after 7 days of bed rest conducted with enteral nutrition provided in either an intermittent (INTERMITTENT) or continuous (CONTINUOUS) pattern (**b**)

As previously reported, GIR declined by ~ 42% following 7 days of bed rest, independent of feeding pattern [data from both groups pooled in the present manuscript; Fig. 6a (Dirks et al. 2019a)]. However, the change in GIR positively correlated with the change in log plasma GLP-1 concentrations (Fig. 6b), but not the change in glucagon, ghrelin or leptin concentrations (all *P* > 0.05).

## Discussion

The present study demonstrates that intermittent enteral nutrition attenuates the increase in post-absorptive plasma insulin and leptin concentrations but has little impact on plasma glucose, GLP-1 and ghrelin concentrations, when compared to continuous enteral nutrition during 7 days of bed rest.

Enteral nutrition is advised when nutritional requirements during hospitalisation are unlikely to be met by oral ingestion for > 5–7 days (Stroud et al. 2003; Singer et al. 2018). Enteral nutrition is generally provided in a continuous pattern (50–100 mL h^−1^ over 12–24 h day^−1^), possibly due to logistics and time commitments of clinical staff, although an intermittent feeding pattern mimicking habitual dietary feeding, is occasionally used (Stroud et al. 2003). The effect of dietary feeding pattern on insulin sensitivity and endocrine responses in humans are not well characterised. This is the first study to demonstrate that the nutrition delivery pattern modulates endocrine responses to enteral nutrition in humans in vivo, during a time period when enteral nutrition is advised (e.g. > 5 days). Our data also demonstrate that intermittent feeding lowers fasting plasma glucose and GLP-1 concentrations compared to continuous feeding, and the changes in fasting GLP-1 are associated with changes in peripheral insulin sensitivity during bed rest.

In addition to peripheral insulin sensitivity, hepatic insulin sensitivity is a major contributor to glucose control (Groop et al. 1989). Hepatic glucose production is primarily regulated by the insulin-to-glucagon ratio (Gonzalez et al. 2016). Here, we demonstrate that insulin concentrations required to maintain euglycemia progressively increase across 7 days of bed rest with continuous enteral feeding, whereas intermittent feeding prevented this increase in insulinemia. Whilst the continuous group would have a continuous exogenous glucose appearance at a rate of ~ 0.2 g min^−1^, this was not sufficient to raise insulinemia until at least 48 h of bed rest, with nutrition provided to meet energy requirements. This suggests that the increase in insulinemia is due to the development of insulin resistance, rather than a physiological response to continuous exogenous carbohydrate appearance. When combined with our previously reported observations that feeding pattern does not alter the decline in peripheral insulin sensitivity or muscle mitochondrial function during bed rest (Dirks et al. 2019a, b), and also with evidence that hepatic insulin sensitivity plays a more important role in glucose control at low insulin concentrations that at high insulin concentrations (Groop et al. 1989), this suggests that hepatic insulin sensitivity was declining with continuous versus intermittent feeding. This is in line with prior observations that 7 days of head down-tilt bed rest with nutritional intake as 3 meals per day, results in decreased peripheral insulin sensitivity, without a decline in hepatic insulin sensitivity in men (Blanc et al. 2000a). Interestingly, women showed decreases in both peripheral and hepatic insulin sensitivity following bed rest (Blanc et al. 2000a). Combined with our data, this suggests that maintaining a degree of fasting between meals can prevent hepatic insulin resistance during bed rest in men, but more work is required to understand if feeding pattern can alter hepatic insulin sensitivity during bed rest in women. Furthermore, our observations require confirmation with the addition of stable-isotope methods to euglycemic–hyperinsulinemic clamps in order to definitively establish whether hepatic insulin sensitivity is altered by enteral feeding pattern during bed rest.

GLP-1 is a gut hormone with a central role in postprandial metabolism (Drucker 2018) and is secreted in response nutrients in the gastrointestinal tract (Gonzalez and Stevenson 2014a; Gonzalez et al. 2015). The present data demonstrate that continuous enteral nutrition delivered at a rate of 100 kcal h^−1^ is insufficient to increase GLP-1 concentrations above those seen in a fasted state. However, following 7 days of bed rest, an intermittent feeding pattern lowered fasting GLP-1 concentrations compared to a continuous feeding pattern. Furthermore, individuals demonstrating the greatest decline in fasting GLP-1 concentrations also demonstrated the greatest decline in glucose infusion rate during a hyperinsulinemic–euglycemic clamp. When considered alongside evidence that exogenous GLP-1 improves glucose homeostasis in ICU patients (Deane et al. 2009), and may enhance glucose disposal in humans (Gutniak et al. 1992; D'Alessio et al. 1994) [although not under all conditions (Orskov et al. 1996; Ahren et al. 1997)], these data suggest that endogenous GLP-1 plays a role in metabolic control during bed rest. As direct effects of GLP-1 on peripheral insulin sensitivity are unclear, the mechanism(s) by which GLP-1 may contribute to metabolic control are thought to also include microvascular recruitment and glucose-stimulated insulin secretion (Drucker 2018). Whilst enteral feeding pattern does not modulate the decline in peripheral insulin sensitivity during bed rest (Dirks et al. 2019a), other strategies that increase endogenous GLP-1 concentrations, such as enteral delivery mode (Luttikhold et al. 2016) and/or nutrient composition (Gonzalez et al. 2015; Gonzalez and Stevenson 2014b; Chen et al. 2019), warrant exploring as potential approaches to preserve GLP1 concentrations during bed rest.

Leptin is a peptide hormone primarily derived from adipose tissue which is mostly known for its role in suppressing appetite, but can also increase insulin sensitivity (Morton and Schwartz 2011), and has been shown to increase during bed rest (Blanc et al. 2000b). Since leptin is thought to be mostly regulated by chronic changes in energy balance, it is important to note that participants in the present study were fed to maintain energy balance, confirmed by the stable fat mass (within 0.1 kg) previously reported (Dirks et al. 2019a). Here, we demonstrate that intermittent feeding lessens the increase plasma leptin concentration seen during bed rest with continuous feeding. This provides the first evidence that feeding pattern can modulate the leptin response to bed rest, independent from energy balance. Since prolonged insulinemia is thought to stimulate increased leptin secretion (Kolaczynski et al. 1996; Gonzalez et al. 2019), the progressive increase in insulinemia seen with continuous vs intermittent feeding may explain this increase in leptin concentrations. If a higher leptin response is desirable, then a continuous feeding pattern could be a strategy to ensure high leptin concentrations independent from changes in energy balance.

Ghrelin is primarily secreted from the gut and is currently the only known gut hormone to stimulate appetite. It has previously been reported that patients in the intensive care unit (ICU) display ~ 50% lower plasma ghrelin concentrations than age- and BMI-matched healthy controls (Nematy et al. 2006). As the present study was conducted on healthy participants, this suggests that reductions in ghrelin concentrations that have previously been reported in the ICU might be a result of physical inactivity per se or changes in feeding mode, rather than illness. It remains to be established whether the reduction in plasma ghrelin concentrations is a direct result of bed rest, naso-gastric feeding, or an interaction between physical inactivity and naso-gastric feeding. Changes in ghrelin concentrations are likely to play a role in appetite regulation during bed rest, as demonstrated by both the positive relationship between appetite and ghrelin concentrations in the present study. This is in line with the positive relationship observed between plasma ghrelin concentrations and ad libitum food intake in ICU patients (Nematy et al. 2006). Since malnutrition is prevalent during and following periods in intensive care, strategies that reduce ghrelin concentrations should be considered with caution. It should also be noted that blood samples and appetite ratings measured during bed rest were taken in in the fasted state for the INTERMITTENT group, compared with constant nutrition in the CONTINUOUS group. Nevertheless, the differences in insulinemia and leptin were progressive, and not apparent within the first 24 h of continuous feeding. This suggests that these responses are a true reflection of the interaction between feeding pattern and bed rest, rather than an artefact of the sampling time point. A further consideration is that we recruited young, healthy men. The generalisability of these findings to clinical nutrition and to women is, therefore, somewhat limited. Nevertheless, by studying healthy people, the present findings are able to isolate the effects of enteral feeding pattern independent from the diverse impact of disease states and injuries seen in clinical settings.

In conclusion, these data demonstrate that enteral feeding pattern alters the endocrine response to bed rest in vivo in humans. Intermittent enteral nutrition attenuates the increase in plasma insulin and leptin concentrations along with the decline in plasma glucagon concentrations during 7 days of bed rest, when compared to a continuous feeding pattern. The increase in insulinemia in the absence of differences in peripheral insulin sensitivity suggests that hepatic insulin sensitivity may be compromised to a greater extent with continuous feeding. If a clinical aim is to attenuate endocrine and metabolic changes seen with enteral feeding during bed rest, then an intermittent feeding pattern may be preferable over continuous delivery of enteral nutrition.

## Abbreviations

GLP-1: Glucagon-like peptide-1
HbA1c: Glycated hemoglobin
RM: Resting metabolic rate
iAUC: Incremental area under the curve
SD: Standard deviation
ANOVA: Analysis of variance
GIR: Glucose infusion rate
